# (2,2′-Bipyridine-κ^2^ *N*,*N*′)[2-*tert*-butyl­anilinato(2−)]dichloridooxido­molybdenum(VI) dichloro­methane hemisolvate

**DOI:** 10.1107/S1600536810047392

**Published:** 2010-11-27

**Authors:** Alastair J. Nielson, Joyce M. Waters

**Affiliations:** a Chemistry, Institute of Natural Sciences, Massey University at Albany, PO Box 102904, North Shore Mail Centre, Auckland, New Zealand

## Abstract

The Mo^VI^ atom in the title structure, [Mo(C_10_H_13_N)Cl_2_O(C_10_H_8_N_2_)]·0.5CH_2_Cl_2_, has a distorted octa­hedral coord­ination sphere with *cis*-orientated oxide and imide ligands, *trans*-chloride ligands and the 2,2′-bipyridine (bipy) ligand N atoms lying *trans* to the oxide and imide ligands. An imide-ligand *tert*-butyl-methyl-group H atom makes a close approach with the oxide ligand (distance = 2.53 Å) and the imide-ligand N atom (distance = 2.41 Å). Another imide-ligand *tert*-butyl-methyl-group H atom makes a close approach to a chloride ligand (distance = 2.82 Å). One bipy-ligand α-H atom makes a close approach to the oxide ligand (distance = 2.4 Å) and the other α-H atom makes a close approach to the imide-ligand phenyl-ring *ortho*-H atom (distance = 2.52 Å). These close approaches suggest the presence of weak intra­molecular hydrogen bonds. The solvent molecule has been modelled under consideration of half-occupancy.

## Related literature

For other oxo-imido complexes, see: Bell *et al.* (1994 ▶); Barrie *et al.* (1999 ▶); Bradley *et al.* (1987 ▶); Clegg *et al.* (1993 ▶); Chatt *et al.* (1979 ▶); Clark *et al.* (1996 ▶,). For the *trans*-influence effect, see: Nugent & Mayer (1988 ▶). For close approaches of hydrogen atoms in transition metal complexes and the relationship to weak hydrogen bonds to oxygen atoms, see: Desiraju (1996 ▶); to chlorine atoms, see: Aakeroy *et al.* (1999 ▶); and to N atoms, see: Demers *et al.* (2005 ▶).
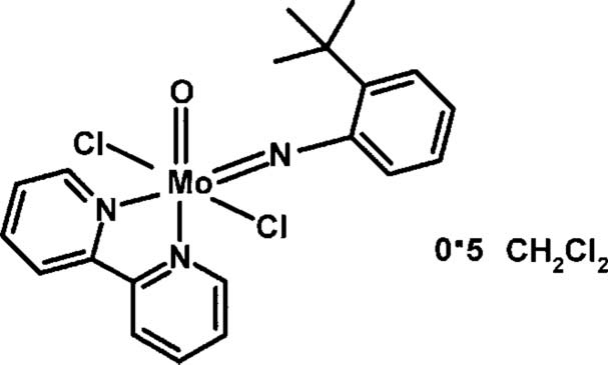


## Experimental

### 

#### Crystal data

[Mo(C_10_H_13_N)Cl_2_O(C_10_H_8_N_2_)]·0.5CH_2_Cl_2_
*M* *_r_* = 528.7
Orthorhombic, 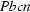

*a* = 17.4207 (2) Å
*b* = 14.9657 (1) Å
*c* = 16.5237 (1) Å
*V* = 4307.94 (6) Å^3^
*Z* = 8
Mo *K*α radiation
μ = 1.00 mm^−1^
*T* = 150 K
0.26 × 0.06 × 0.06 mm

#### Data collection

Siemens SMART diffractometer
Absorption correction: multi-scan (Blessing, 1995 ▶) *T*_min_ = 0.651, *T*_max_ = 0.963
40908 measured reflections
4459 independent reflections
3438 reflections with *I* > 2σ(*I*)
*R*_int_ = 0.084

#### Refinement

*R*[*F* ^2^ > 2σ(*F* ^2^)] = 0.043
*wR*(*F* ^2^) = 0.102
*S* = 1.03
4459 reflections
280 parameters
H atoms treated by a mixture of independent and constrained refinement
Δρ_max_ = 0.64 e Å^−3^
Δρ_min_ = −1.54 e Å^−3^

### 

Data collection: *SMART* (Siemens, 1995 ▶); cell refinement: *SAINT* (Siemens, 1995 ▶); data reduction: *SAINT*; program(s) used to solve structure: *SHELXS97* (Sheldrick, 2008 ▶); program(s) used to refine structure: *SHELXL97* (Sheldrick, 2008 ▶); molecular graphics: *ORTEP-3 for Windows* (Farrugia, 1999 ▶); software used to prepare material for publication: *SHELXL97*.

## Supplementary Material

Crystal structure: contains datablocks I, global. DOI: 10.1107/S1600536810047392/bv2152sup1.cif

Structure factors: contains datablocks I. DOI: 10.1107/S1600536810047392/bv2152Isup2.hkl

Additional supplementary materials:  crystallographic information; 3D view; checkCIF report

## Figures and Tables

**Table 1:** Hydrogen-bond geometry (Å, °)

*D*—H⋯*A*	*D*—H	H⋯*A*	*D*⋯*A*	*D*—H⋯*A*
C18—H18*A*⋯O1	0.98	2.53	3.492 (4)	167
C18—H18*A*⋯N1	0.98	2.41	3.070 (5)	124
C19—H19*C*⋯Cl1	0.98	2.82	3.762 (4)	162
C19—H19*C*⋯N1	0.98	2.39	3.036 (5)	123

**Fig. 1.**
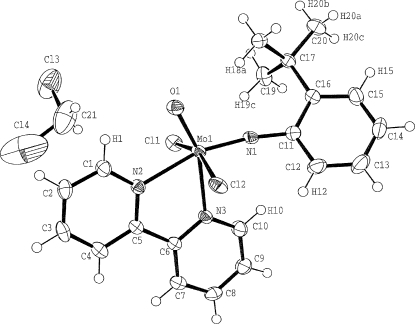
ORTEP diagram, at the 50% probability level, of the mlecule showing the numbering system.

## supplementary crystallographic information

### Comment

Complexes of molybdenum containing oxo and imido functions in the same molecule are still fairly rare. However they are relatively easy to prepare by a conproportionation reaction between bis-imido complexes of the form [Mo(NR)_2_Cl_2_(dme)] (dme =1,2- dimethoxyethane) and the bis-oxo complexes [Mo(O)_2_Cl_2_(dme)] (Bell *et al.*, 1994). During attempts to prepare bis-imido complexes in which the imido ligand carried substituents on the aryl ring possessing potentially sterically hindering *ortho*-substituents, we reacted Na_2_MoO_4_ with two equivalents of 2-*tert*-butylaniline in the presence of 8 equivalents of SiMe_3_Cl and 4 equivalents of NEt_3_ in dme as solvent which is the normal protocol for producing [Mo(NR)_2_Cl_2_(dme)] complexes in good yield. A red solid was obtained which had the characteristic features of the complex. This complex was then reacted with 2,2'-bipyridine (bipy) to produce the complex [Mo(NC_6_H_4_CMe_3_-2)_2_Cl_2_(bipy)]. This type of complex was of interest as our studies of bis-imido tungsten complexes of the form [W(NC_6_H_5_)_2_Cl_2_(bipy)] had shown there was a steric interaction between the bipy α-H atoms and the *ipso*-carbon of the phenyl ring (Bradley *et al.*, 1987). Whereas the imido ligand nitrogen atoms were bent away from each other as expected, the *ipso*-carbon atoms of the phenyl were bent inwards towards each other [W—N—C bond angles 165.6 (12) and 164.4 (12)°] apparently to reduce contact with the bipy α-H atoms. The imido ligand phenyl groups were also apparently rotated to reduce contact of the *ortho*-H atoms with the bipy α-H atoms. The product obtained from the reaction with bipy crystallized nicely but did not give particularly good C, H and N analytical data. The NMR spectra suggested the bulk sample was indeed [Mo(NC_6_H_4_CMe_3_-2)_2_Cl_2_(bipy)] but the spectra also indicated a small amount of a second species was present. A crystal picked out from the mass and subjected to an X-ray analysis was found not to be the bis-imido complex but instead the oxo-imido complex [Mo (NC_6_H_4_CMe_3_-2)(O)Cl_2_(bipy)](1). The oxo-imido function could have arisen as a by-product during the preparation of [Mo(NC_6_H_4_CMe_3_ 2)_2_Cl_2_(dme)] by incomplete oxo-imido exchange or by hydrolysis of one of the imido functions during the exchange of the dme ligand for the bipy ligand as this ligand was not dried after obtaining it from commercial sources.

The structure of (1) consists of a distorted-octahedral array about the molybdenum atom with a *cis*-orientation of the organoimido and oxo ligands, *trans*-chloro ligands and the nitrogen atoms of the bipyridyl ligand lying *trans* to the organoimido and oxo ligands (Fig. 1). The overall structure is similar to that observed for the tungsten-bipy complex [WCl_2_(NCMe_3_)(O)(bipy)] (Clegg *et al.*, 1993) and also the molybdenum complexes [MoCl_2_(NH)(O)(OPPh_2_Et)] (Chatt *et al.* 1979) and [MoCl_2_(NC_6_H_2_Ph_3_-2,4,6)(O)(dme)] (Clark *et al.* 1996). The Mo—N_imido_ bond length [1.735 (3) Å] and Mo—O_oxo_ bond length [1.686 (2) Å] are similar to those found in [MoCl_2_(NC_6_H_2_Ph_3_-2,4,6)(O)(dme)] [1.756 (7) and 1.700 (6) Å] (Clark *et al.* 1996). The Mo—Cl(1) bond length [2.3580 (9) Å] is slightly shorter than the Mo—Cl(2) bond length [2.3745 (9) Å]. The longer Mo—Cl bond length is similar in distance to the shorter Mo—Cl bond in [MoCl_2_(NC_6_H_2_Ph_3_-2,4,6)(O)(dme)] [2.375 (3) Å] but both bonds are shorter than those found in [MoCl_2_(NH)(O)(OPPh_2_Et)] [average 2.391 (7) Å] or the *bis*-imido complex [MoCl_2_(NC_10_H_15_)(NC_6_F_5_)(dme)] (C_10_H_15 _= adamantyl) [average 2.397 (2) Å] (Bell *et al.* 1994). The Mo—N bond *trans* to the oxo ligand is slightly longer than that *trans* to the imido function [2.283 (3) and 2.255 (3) Å respectively] which suggests that the oxo ligand may exert the stronger *trans* -influence (Nugent & Mayer, 1988). However this may not be a *trans* -influence effect as there are close approaches of the two α-hydrogen atoms of the bipy ligand with other parts of the molecule.

The N(1)—Mo—O(1) bond angle [104.7 (1)°] and Cl(1)—Mo—Cl(2) bond angle [160.08 (3)°] in (1) are similar to those found in [MoCl_2_(NC_6_H_2_Ph_3_-2,4,6)(O)(dme)] [bond angles 104.2 (4) and 159.7 (1)° respectively] and the two chloro ligands push away from both the Mo—O(1) and Mo—N(1) multiple bonds. In this respect, the O(1)—Mo—Cl(1) and O(1)—Mo—Cl(2) bond angles (oxo ligand and chloro ligands) are essentially equivalent [96.83 (8) and 97.41 (8)° respectively] but the N(1)—Mo—Cl(1) angle [99.78 (9)°] is greater than the N(1)—Mo—Cl(2) angle [90.00 (9)°] and this appears to be related to a steric effect arising from the proximity of the 2-*tert*-butyl substituent on the imido ligand aryl ring (see later). The angles associated with the coordinated nitrogen atoms of the bipy rings show there is nothing unusual for the way this ligand coordinates to the metal with the O(1)—Mo—N(3), N(1)—Mo—N(2) and N(2)—Mo—N(3) bond angles [159.2 (1), 164.4 (1) and 69.97 (9)° respectively] being similar to those found for [WCl_2_(NCMe_3_)(O)(bipy)] [157.9 (2), 165.9 (2) and 69.1 (2)° respectively] (Clegg *et al.*, 1993) or for the *bis*-imido tungsten complex [WCl_2_(NPh)_2_(bipy)] [161.2 (5), 164.3 (5) and 69.8 (4)° respectively] (Bradley *et al.* 1987). These angles do not differ significantly from those associated with the coordination mode of the 1,2-dimethoxyethane ligand in [MoCl_2_(NC_6_H_2_Ph_3_-2,4,6)(O)(dme)]- [O_oxo_—Mo—O, N_imido_—Mo—O and O—Mo—O angles 160.3 (3), 165.9 (3) and 70.6 (2) respectively]. The coordinated bipy ligand is essentially planar with the difference between the two planes made by the two rings being only 3.9 (2)°.

The phenyl ring of the organoimido ligand is bent back towards Cl(2) and also N(3) of the bipy ring with the Mo—N(1)—C(11) bond angle being 165.8 (2)\ which is smaller than in [MoCl_2_(NC_6_H_2_Ph_3_-2,4,6)(O)(dme)] [172.2 (7)°] (Clark *et al.*, 1996) and the tungsten bipy analogue [WCl_2_(NCMe_3_)(O)(bipy)] [170.6 (5)°] (Clegg *et al.*, 1993) but similar to that found in the *bis*-imido complexes [Mo(NC_6_H_3_Cl_2_-2,6)(S_2_CNEt_2_)_2_][162.2 (7) and 162.9 (7)/%] (Barrie *et al.*, 1999) and [WCl_2_(NPh)_2_(bipy)] [165.6 (12) and 164.4 (11)°] (Bradley *et al.*, 1987). However in these complexes the M—N—C bond angles are such that the phenyl or alkyl group bends in towards the adjacent oxo or imido ligand whereas in the present complex the bend is away from the oxo ligand. This appears to be caused by the *tert*-butyl substituent which in the crystal prefers to orientate over Cl(1) and O(1) rather than Cl(2) and O(1) which appears to be another possible orientation (Fig 1). The rotation of the organoimido phenyl ring is such that the plane of the ring deviates from the plane made by the bipy rings by 46.5 (1)° (Fig 1). The orientation of the phenyl ring and the orientation of the 2-*tert*-butyl substituent has some interesting consequences for intramolecular contacts. For the 2-*tert*-butyl substituent the rotation about C(16) and C(17) is such that one of the equatorially positioned methyl groups [C(18)] lies directly above the oxo ligand and H(18*a*) makes a contact of 2.53 Å with it. This distance lies within the range of distances considered to involve weak hydrogen bonding to oxygen atoms (Desiraju, 1996)). There is an even shorter separation of 2.41 Å between H(18) and the imido nitrogen atom, N(1) and the separation is well within the range of values considered to involve weak hydrogen bonding to nitrogen [2.65%A (Demers *et al.*, 2005)]. It is interesting to note that even though the nitrogen lone pair will be mostly involved in donation to molybdenum to make the imido ligand multiple bond, the bend made by the Mo—N(1)—C(11) system [165.8 (2)°] is such that any remaining lone pair is pointing in the direction of H(18*a*). The other equatorially positioned methyl group of the 2-*tert*-butyl substituent lies above Cl(1) with the H(19*c*) to Cl(1) separation of 2.82 Å. This distance is also within the range of values suggested as weak hydrogen bonding to chlorine (Aakeroy *et al.* 1999). The separation between H(19*c*) and the imido nitrogen N(1) is 2.39Å which suggests potential weak hydrogen bonding may also be involved. There is a similar approach of H(18*a*) to N1 (2.41 Å). However it should be realised that these close approaches are forced on the system by the molecular geometry of the *tert*-butyl group which may or may not imply the existence of attractive H-bonding. The remaining methyl group of the 2-*tert*-butyl substituent, which lies in an axial position, is rotated to give a gearing effect which removes any interaction of the H atoms with the nearest neighbour H atoms. Thus H(20*a*) is positioned so as to bisect the C(18)—C(17)—C(19) angle and this allows H(20*b*) and H(20*c*) to lie in front of, but to either side of, the bipy α-hydrogen H(15). As a result of the positioning of the 2-*tert*-butyl substituent, H(12), which lies in the other *ortho*-position of the aromatic ring, makes a close contact with Cl(2) with the distance of 3.06 Å being just outside the limit of the H and Cl van der Waals radii (3.0 Å) but still representing a weak hydrogen bond (Aakeroy, 1999). H(12) also makes a close contact of 2.52 Å with the α-hydrogen of the nearby bipy ring which is just outside the van der waals radii of 2.4 Å (Aakeroy, 1999). On the other side of the molecule there is a close approach of the weak hydrogen bonding type, for H(1) which is the other α-hydrogen of the bipy ring, with the terminal oxo ligand oxygen [O(1)]. This arises since the bipy rings are essentially co-planar with the Mo—O multiple bond. The atomic separation is 2.44 Å which is even shorter than the distance the 2-*tert*-butyl substituent H(18*a*) atom makes with O(1) (2.53 Å). The separation for this type of interaction in [WCl_2_(NCMe_3_)(O)(bipy)] is 2.42 Å (Clegg, *et al.*, 1993). There are no other significant intramolecular contacts in the structure. The disordered partial CH_2_Cl_2_ molecule lies across a centre of symmetry in the crystal lattice but its H atoms make no significant approaches to the chlorine complex.

### Experimental

Na_2_MoO_4_.2H_2_O (2.0 g, 8.3 mmol) was dried under vacuum by heating at 100°C for 1 h to yield anhydrous Na_2_MoO_4_ (1.7 g, 8.3 mmol). 2-*tert*-butylaniline (2.46 g, 16.5 mmol) was added followed by 1,2-dimethoxyethane (50 cm^3^) and then triethylamine (4.6 cm^3^ 33.0 mmol) and the mixture was stirred rapidly while chlorotrimethylsilane (8.4 cm^3^ 66.0 mmol) was added dropwise. The mixture was stirred for 16 h, refluxed for 8 h and then filtered while the mixture was still hot and the solvent removed to give a deep red crystalline solid (4.43 g). 0.76 g of this material was added to 2,2'-bipyridine (0.214 g, 1.4 mmol), CH_2_Cl_2_ (30 cm^3^) was added and the mixture stirred for 5 h. The solution was filtered, the solvent removed and the deep-red crystalline solid washed with petroleum spirit. The solid was dissolved in CH_2_Cl_2_ (20 cm^3^) the volume reduced to *ca* one-half and the solution allowed to stand at room temperature yielding a red-brown crystalline solid (0.55 g). A crystal was chosen from the mass and the X-ray crystal structure obtained.

### Refinement

H atoms were placed in calculated positions riding on the atoms to which they are attached. The CH_2_Cl_2_ solvent was located close to a centre of symmetry requiring that it be no more than half-weighted. No attempt was made to refine its site occupancy factor.

### Crystal data

**Table:**

[Mo(C_10_H_13_N)Cl_2_O(C_10_H_8_N_2_)]·0.5CH_2_Cl_2_	*F*(000) = 2136
*M**_r_* = 528.7	*D*_x_ = 1.630 Mg m^−^^3^
Orthorhombic, *P**b**c**n*	Mo *K*α radiation, λ = 0.71073 Å
Hall symbol: -P 2n 2ab	Cell parameters from 8192 reflections
*a* = 17.4207 (2) Å	θ = 2–25°
*b* = 14.9657 (1) Å	µ = 1.00 mm^−^^1^
*c* = 16.5237 (1) Å	*T* = 150 K
*V* = 4307.94 (6) Å^3^	Needle, yellow
*Z* = 8	0.26 × 0.06 × 0.06 mm

### Data collection

**Table:**

Siemens SMART diffractometer	4459 independent reflections
Radiation source: fine-focus sealed tube	3438 reflections with *I* > 2σ(*I*)
graphite	*R*_int_ = 0.084
Area detector ω scan	θ_max_ = 26.6°, θ_min_ = 1.8°
Absorption correction: multi-scan (Blessing, 1995)	*h* = 0→21
*T*_min_ = 0.651, *T*_max_ = 0.963	*k* = 0→18
40908 measured reflections	*l* = 0→20

### Refinement

**Table:**

Refinement on *F*^2^	Primary atom site location: structure-invariant direct methods
Least-squares matrix: full	Secondary atom site location: difference Fourier map
*R*[*F*^2^ > 2σ(*F*^2^)] = 0.043	Hydrogen site location: inferred from neighbouring sites
*wR*(*F*^2^) = 0.102	H atoms treated by a mixture of independent and constrained refinement
*S* = 1.03	*w* = 1/[σ^2^(*F*_o_^2^) + (0.054*P*)^2^ + 1.7768*P*] where *P* = (*F*_o_^2^ + 2*F*_c_^2^)/3
4459 reflections	(Δ/σ)_max_ = 0.008
280 parameters	Δρ_max_ = 0.64 e Å^−^^3^
0 restraints	Δρ_min_ = −1.54 e Å^−^^3^

### Special details

**Table:**

Geometry. All e.s.d.'s (except the e.s.d. in the dihedral angle between two l.s. planes) are estimated using the full covariance matrix. The cell e.s.d.'s are taken into account individually in the estimation of e.s.d.'s in distances, angles and torsion angles; correlations between e.s.d.'s in cell parameters are only used when they are defined by crystal symmetry. An approximate (isotropic) treatment of cell e.s.d.'s is used for estimating e.s.d.'s involving l.s. planes.
Refinement. Refinement of *F*^2^ against ALL reflections. The weighted *R*-factor *wR* and goodness of fit *S* are based on *F*^2^, conventional *R*-factors *R* are based on *F*, with *F* set to zero for negative *F*^2^. The threshold expression of *F*^2^ > σ(*F*^2^) is used only for calculating *R*-factors(gt) *etc*. and is not relevant to the choice of reflections for refinement. *R*-factors based on *F*^2^ are statistically about twice as large as those based on *F*, and *R*- factors based on ALL data will be even larger.

### Fractional atomic coordinates and isotropic or equivalent isotropic displacement parameters (Å^2^)

**Table:**

	*x*	*y*	*z*	*U*_iso_*/*U*_eq_	Occ. (<1)
Mo1	0.353791 (15)	0.84420 (2)	0.150599 (16)	0.02018 (11)	
Cl1	0.31084 (5)	0.72649 (6)	0.06725 (5)	0.0299 (2)	
Cl2	0.41115 (5)	0.98134 (6)	0.19018 (5)	0.0291 (2)	
N1	0.26663 (15)	0.87982 (19)	0.18979 (16)	0.0208 (6)	
N2	0.46431 (15)	0.83274 (18)	0.08033 (16)	0.0218 (6)	
N3	0.34325 (15)	0.91836 (19)	0.03034 (16)	0.0226 (6)	
O1	0.39213 (13)	0.77987 (16)	0.22394 (13)	0.0257 (5)	
C1	0.52275 (19)	0.7863 (2)	0.1095 (2)	0.0269 (8)	
H1	0.5178	0.7587	0.1610	0.032*	
C2	0.5900 (2)	0.7769 (3)	0.0679 (2)	0.0298 (8)	
H2	0.6311	0.7430	0.0900	0.036*	
C3	0.5975 (2)	0.8169 (3)	−0.0060 (2)	0.0300 (8)	
H3	0.6436	0.8111	−0.0362	0.036*	
C4	0.53740 (19)	0.8656 (2)	−0.0355 (2)	0.0258 (8)	
H4	0.5416	0.8943	−0.0865	0.031*	
C5	0.47099 (19)	0.8728 (2)	0.00858 (18)	0.0197 (7)	
C6	0.40356 (18)	0.9217 (2)	−0.01916 (18)	0.0197 (7)	
C7	0.4003 (2)	0.9672 (2)	−0.0908 (2)	0.0258 (8)	
H7	0.4441	0.9698	−0.1250	0.031*	
C8	0.3339 (2)	1.0088 (2)	−0.1132 (2)	0.0282 (8)	
H8	0.3311	1.0415	−0.1623	0.034*	
C9	0.2717 (2)	1.0023 (3)	−0.0635 (2)	0.0327 (9)	
H9	0.2243	1.0289	−0.0784	0.039*	
C10	0.2782 (2)	0.9575 (3)	0.0072 (2)	0.0300 (8)	
H10	0.2347	0.9539	0.0417	0.036*	
C11	0.20734 (19)	0.9275 (2)	0.22186 (19)	0.0225 (7)	
C12	0.2068 (2)	1.0188 (2)	0.2053 (2)	0.0341 (9)	
H12	0.2446	1.0435	0.1705	0.041*	
C13	0.1527 (2)	1.0727 (3)	0.2388 (3)	0.0425 (10)	
H13	0.1533	1.1352	0.2290	0.051*	
C14	0.0974 (2)	1.0355 (3)	0.2866 (3)	0.0415 (10)	
H14	0.0589	1.0725	0.3097	0.050*	
C15	0.0967 (2)	0.9464 (3)	0.3014 (2)	0.0327 (9)	
H15	0.0568	0.9227	0.3341	0.039*	
C16	0.15120 (18)	0.8890 (2)	0.27117 (19)	0.0219 (7)	
C17	0.15032 (18)	0.7900 (2)	0.2892 (2)	0.0241 (7)	
C18	0.2222 (2)	0.7626 (3)	0.3349 (2)	0.0294 (8)	
H18A	0.2676	0.7779	0.3028	0.044*	
H18B	0.2212	0.6980	0.3446	0.044*	
H18C	0.2241	0.7941	0.3868	0.044*	
C19	0.1439 (2)	0.7378 (3)	0.2113 (2)	0.0324 (9)	
H19A	0.0978	0.7567	0.1820	0.049*	
H19B	0.1404	0.6738	0.2236	0.049*	
H19C	0.1893	0.7489	0.1778	0.049*	
C20	0.0821 (2)	0.7650 (3)	0.3419 (2)	0.0374 (10)	
H20A	0.0853	0.7971	0.3935	0.056*	
H20B	0.0826	0.7005	0.3520	0.056*	
H20C	0.0344	0.7813	0.3141	0.056*	
C21	0.4547 (10)	0.5417 (12)	0.0336 (9)	0.088 (5)	0.50
H21A	0.428 (7)	0.558 (9)	0.007 (7)	0.080*	0.50
H21B	0.452 (7)	0.562 (8)	0.074 (7)	0.080*	0.50
Cl3	0.4319 (3)	0.4302 (3)	0.0347 (4)	0.0822 (14)	0.50
Cl4	0.5425 (4)	0.5541 (6)	−0.0196 (6)	0.162 (4)	0.50

### Atomic displacement parameters (Å^2^)

**Table:**

	*U*^11^	*U*^22^	*U*^33^	*U*^12^	*U*^13^	*U*^23^
Mo1	0.01602 (16)	0.02499 (18)	0.01954 (16)	−0.00289 (12)	0.00123 (11)	0.00364 (12)
Cl1	0.0277 (5)	0.0339 (5)	0.0282 (4)	−0.0058 (4)	−0.0006 (3)	−0.0033 (4)
Cl2	0.0271 (4)	0.0320 (5)	0.0281 (4)	−0.0105 (4)	0.0059 (4)	−0.0008 (4)
N1	0.0183 (14)	0.0206 (15)	0.0234 (14)	−0.0059 (11)	0.0018 (11)	0.0032 (12)
N2	0.0175 (14)	0.0252 (16)	0.0226 (14)	−0.0013 (12)	−0.0004 (11)	−0.0001 (12)
N3	0.0173 (14)	0.0274 (16)	0.0230 (14)	−0.0006 (12)	0.0021 (11)	0.0040 (12)
O1	0.0206 (12)	0.0298 (14)	0.0267 (12)	−0.0048 (11)	−0.0016 (9)	0.0044 (10)
C1	0.0205 (18)	0.034 (2)	0.0264 (18)	0.0011 (15)	−0.0027 (14)	0.0007 (16)
C2	0.0201 (18)	0.038 (2)	0.0318 (19)	0.0040 (16)	−0.0035 (15)	−0.0048 (16)
C3	0.0172 (18)	0.037 (2)	0.036 (2)	−0.0023 (16)	0.0038 (15)	−0.0102 (17)
C4	0.0251 (18)	0.030 (2)	0.0217 (17)	−0.0070 (15)	0.0037 (14)	−0.0045 (14)
C5	0.0205 (17)	0.0202 (17)	0.0185 (16)	−0.0042 (14)	0.0007 (13)	−0.0035 (13)
C6	0.0213 (17)	0.0194 (17)	0.0185 (15)	−0.0040 (13)	0.0015 (13)	−0.0022 (13)
C7	0.0291 (19)	0.0259 (19)	0.0223 (17)	−0.0049 (16)	0.0030 (14)	−0.0010 (14)
C8	0.038 (2)	0.026 (2)	0.0207 (17)	−0.0007 (16)	−0.0037 (15)	0.0039 (15)
C9	0.0254 (18)	0.039 (2)	0.0340 (19)	0.0058 (17)	−0.0035 (16)	0.0096 (16)
C10	0.0212 (18)	0.039 (2)	0.0294 (19)	0.0022 (17)	−0.0004 (15)	0.0065 (16)
C11	0.0191 (17)	0.0248 (19)	0.0234 (17)	−0.0022 (14)	−0.0032 (13)	0.0015 (14)
C12	0.036 (2)	0.025 (2)	0.041 (2)	−0.0031 (17)	0.0010 (17)	0.0089 (17)
C13	0.049 (3)	0.022 (2)	0.057 (3)	0.0062 (19)	−0.001 (2)	0.0035 (19)
C14	0.038 (2)	0.034 (2)	0.053 (3)	0.0149 (19)	0.0056 (19)	−0.0036 (19)
C15	0.0252 (19)	0.039 (2)	0.034 (2)	0.0035 (17)	0.0060 (16)	−0.0007 (17)
C16	0.0199 (17)	0.0242 (18)	0.0215 (16)	−0.0030 (14)	−0.0010 (13)	−0.0007 (14)
C17	0.0193 (17)	0.0249 (19)	0.0279 (18)	−0.0042 (14)	0.0045 (14)	0.0030 (14)
C18	0.0279 (19)	0.031 (2)	0.0293 (19)	0.0032 (16)	0.0030 (15)	0.0080 (15)
C19	0.0224 (19)	0.031 (2)	0.044 (2)	−0.0076 (16)	0.0045 (16)	−0.0087 (17)
C20	0.030 (2)	0.038 (2)	0.044 (2)	−0.0088 (18)	0.0117 (17)	0.0087 (18)
C21	0.096 (11)	0.084 (11)	0.085 (11)	0.034 (9)	−0.031 (8)	−0.035 (9)
Cl3	0.054 (2)	0.065 (3)	0.127 (3)	−0.001 (2)	−0.021 (2)	−0.001 (3)
Cl4	0.123 (6)	0.162 (6)	0.200 (7)	−0.087 (5)	−0.108 (5)	0.089 (5)

### Geometric parameters (Å, °)

**Table:**

Mo1—O1	1.686 (2)	C12—C13	1.359 (5)
Mo1—N1	1.735 (3)	C12—H12	0.9500
Mo1—N2	2.255 (3)	C13—C14	1.364 (6)
Mo1—N3	2.283 (3)	C13—H13	0.9500
Mo1—Cl1	2.3580 (9)	C14—C15	1.356 (5)
Mo1—Cl2	2.3745 (9)	C14—H14	0.9500
N1—C11	1.363 (4)	C15—C16	1.375 (5)
N2—C1	1.323 (4)	C15—H15	0.9500
N2—C5	1.334 (4)	C16—C17	1.511 (5)
N3—C10	1.332 (4)	C17—C19	1.510 (5)
N3—C6	1.332 (4)	C17—C18	1.519 (5)
C1—C2	1.366 (5)	C17—C20	1.520 (4)
C1—H1	0.9500	C18—H18A	0.9800
C2—C3	1.365 (5)	C18—H18B	0.9800
C2—H2	0.9500	C18—H18C	0.9800
C3—C4	1.365 (5)	C19—H19A	0.9800
C3—H3	0.9500	C19—H19B	0.9800
C4—C5	1.371 (4)	C19—H19C	0.9800
C4—H4	0.9500	C20—H20A	0.9800
C5—C6	1.458 (4)	C20—H20B	0.9800
C6—C7	1.367 (4)	C20—H20C	0.9800
C7—C8	1.363 (5)	C21—Cl4^i^	1.453 (17)
C7—H7	0.9500	C21—Cl3	1.715 (19)
C8—C9	1.363 (5)	C21—Cl4	1.77 (2)
C8—H8	0.9500	C21—H21A	0.70 (12)
C9—C10	1.350 (5)	C21—H21B	0.74 (11)
C9—H9	0.9500	Cl3—Cl4^i^	0.563 (9)
C10—H10	0.9500	Cl4—Cl3^i^	0.563 (9)
C11—C12	1.393 (5)	Cl4—C21^i^	1.453 (17)
C11—C16	1.397 (4)	Cl4—Cl4^i^	2.287 (12)
			
O1—Mo1—N1	104.71 (12)	C9—C10—H10	118.7
O1—Mo1—N2	89.34 (10)	N1—C11—C12	116.2 (3)
N1—Mo1—N2	164.37 (11)	N1—C11—C16	122.8 (3)
O1—Mo1—N3	159.24 (10)	C12—C11—C16	121.0 (3)
N1—Mo1—N3	96.03 (11)	C13—C12—C11	120.5 (4)
N2—Mo1—N3	69.97 (9)	C13—C12—H12	119.8
O1—Mo1—Cl1	96.83 (8)	C11—C12—H12	119.8
N1—Mo1—Cl1	99.78 (9)	C12—C13—C14	118.9 (4)
N2—Mo1—Cl1	85.03 (7)	C12—C13—H13	120.5
N3—Mo1—Cl1	80.17 (7)	C14—C13—H13	120.5
O1—Mo1—Cl2	97.41 (8)	C15—C14—C13	120.8 (4)
N1—Mo1—Cl2	90.00 (9)	C15—C14—H14	119.6
N2—Mo1—Cl2	81.28 (7)	C13—C14—H14	119.6
N3—Mo1—Cl2	81.57 (7)	C14—C15—C16	122.9 (4)
Cl1—Mo1—Cl2	160.08 (3)	C14—C15—H15	118.5
C11—N1—Mo1	165.8 (2)	C16—C15—H15	118.5
C1—N2—C5	119.5 (3)	C15—C16—C11	115.9 (3)
C1—N2—Mo1	120.6 (2)	C15—C16—C17	122.3 (3)
C5—N2—Mo1	119.9 (2)	C11—C16—C17	121.8 (3)
C10—N3—C6	118.5 (3)	C19—C17—C16	109.9 (3)
C10—N3—Mo1	122.2 (2)	C19—C17—C18	110.2 (3)
C6—N3—Mo1	119.3 (2)	C16—C17—C18	110.7 (3)
N2—C1—C2	122.0 (3)	C19—C17—C20	107.6 (3)
N2—C1—H1	119.0	C16—C17—C20	111.2 (3)
C2—C1—H1	119.0	C18—C17—C20	107.1 (3)
C3—C2—C1	119.1 (3)	C17—C18—H18A	109.5
C3—C2—H2	120.5	C17—C18—H18B	109.5
C1—C2—H2	120.5	H18A—C18—H18B	109.5
C4—C3—C2	118.7 (3)	C17—C18—H18C	109.5
C4—C3—H3	120.6	H18A—C18—H18C	109.5
C2—C3—H3	120.6	H18B—C18—H18C	109.5
C3—C4—C5	120.0 (3)	C17—C19—H19A	109.5
C3—C4—H4	120.0	C17—C19—H19B	109.5
C5—C4—H4	120.0	H19A—C19—H19B	109.5
N2—C5—C4	120.7 (3)	C17—C19—H19C	109.5
N2—C5—C6	115.8 (3)	H19A—C19—H19C	109.5
C4—C5—C6	123.5 (3)	H19B—C19—H19C	109.5
N3—C6—C7	121.2 (3)	C17—C20—H20A	109.5
N3—C6—C5	115.0 (3)	C17—C20—H20B	109.5
C7—C6—C5	123.8 (3)	H20A—C20—H20B	109.5
C8—C7—C6	119.8 (3)	C17—C20—H20C	109.5
C8—C7—H7	120.1	H20A—C20—H20C	109.5
C6—C7—H7	120.1	H20B—C20—H20C	109.5
C7—C8—C9	118.6 (3)	Cl3—C21—Cl4	107.9 (9)
C7—C8—H8	120.7	Cl4^i^—C21—H21A	106 (10)
C9—C8—H8	120.7	Cl3—C21—H21A	101 (10)
C10—C9—C8	119.3 (3)	Cl4—C21—H21A	103 (10)
C10—C9—H9	120.4	Cl4^i^—C21—H21B	123 (10)
C8—C9—H9	120.4	Cl3—C21—H21B	112 (10)
N3—C10—C9	122.6 (3)	Cl4—C21—H21B	118 (10)
N3—C10—H10	118.7	H21A—C21—H21B	113 (10)

Symmetry codes: (i) −*x*+1, −*y*+1, −*z*.

### Hydrogen-bond geometry (Å, °)

**Table:**

*D*—H···*A*	*D*—H	H···*A*	*D*···*A*	*D*—H···*A*
C18—H18A···O1	0.98	2.53	3.492 (4)	167
C18—H18A···N1	0.98	2.41	3.070 (5)	124
C19—H19C···Cl1	0.98	2.82	3.762 (4)	162
C19—H19C···N1	0.98	2.39	3.036 (5)	123
